# Woman Varayoc of Peruvian Andes

**DOI:** 10.3389/fsoc.2023.1232615

**Published:** 2023-11-16

**Authors:** Edgar Gutiérrez-Gómez, Jesús Wiliam Huanca-Arohuanca, Adolfo Quispe-Arroyo, Rosa Cecilia González-Ríos, Yodel Cheldo Huari-Salazar

**Affiliations:** ^1^Facultad de Ingeniería y Gestión, Escuela de Administración de Turismo Sostenible y Hotelería, Universidad Nacional Autónoma de Huanta, Ayacucho, Peru; ^2^Universidad Nacional de San Agustín de Arequipa, Arequipa, Peru; ^3^Departamento de Académico de Educación y Ciencias Humanas, Facultad de Ciencias de la Educación, Universidad Nacional de San Cristóbal de Huamanga, Ayacucho, Peru

**Keywords:** Andean woman, Andes, power, symbolism, woman

## Abstract

This study focuses on the importance of the symbolism of social control by the woman as the Varayoc (an office of authority of Incan origin) in a community in the Peruvian Andes. The objective is to explain the survival of the office of the Varayoc—traditionally held exclusively by men—and the acceptance of Andean women to it as a recognition of gender equality. In this field investigation, we interviewed and observed the most important activities of a woman Varayoc administering justice and present in all communal tasks. We conclude that women in the Peruvian Andes are approaching a status of equality with men in their position as the Varayoc, while maintaining the Incan tradition of local governance through the symbolism of the rod of command, which is also called Varayoc. It is evident that more women in the Andean community are interested in assuming political leadership with the symbolism of the ancestral Varayoc.

## 1. Introduction

The research for this study was conducted in the Ccarhuaccocco community that is located in Paras, a district in Cangallo province belonging to the Department and Region of Ayacucho in Peru. Located in the Andes Mountains, Paras has an altitude of 3,739 m. The Andes divide the territory of Peru into three natural regions: the coast, the highlands, and the jungle. A certain form of Incan political administration is still in effect in some populations of the highlands. Owing to its remote geographical location as well as its difficulty of access from the administrative and geopolitical center of Lima, the capital city of Peru, Ccarhuaccocco maintains the ancient Incan tradition of administration by the Varayoc. The latter is an official who uses a rod, which is a symbol of social control over the people: “The Varayoc uses an ornate and decorated rod as a symbol of command. Normally, the indigenous communities [in the past] nominated as the Varayoc the oldest person, who exercised their authority firmly, with justice and equity” (Zavaleta, 2019). Whoever carries the rod and exemplifies its function of social administration is called the Varayoc or *envarado.*^1^ It symbolizes their authority over the entire population and the area. The Varayoc is in charge of managing the residents of their community and receiving visitors.

Owing to its high altitude and geographical conditions, agriculture and livestock constitute the economic livelihood of the residents of Ccarhuaccocco. In light of the vulnerability of the Andean economy, the regional government of Ayacucho and the mayor of Paras agreed to promote Ccarhuaccocco as a tourist destination, and emphasized the importance of *Apu Ritipata* and *Ñaupallaqta Qachir,* which are “are two sites from the Ccarhuaccocco community, as well as colonial-era bridges in the region. In addition, the Regional Government of Ayacucho commissioned the department in charge of economic development to identify archaeological sites in the area” (Redacción, 2023). This tourism initiative led to an unexpected visit by the research team to the community of Ccarhuaccocco to corroborate the local tourist sites that were being promoted, such as the *Apu Ritipata,* the highest snow-covered mountain in the region (4,900 m) that is also considered sacred, and the *Ñaupallaqta Qachir, −*a pre-Inca archaeological site. The field visit yielded an important insight that had been omitted in tourism-centric advertisements of the region and its culture: the survival of the symbolism of social control exercised by the Varayoc, especially the fact that the bearer of the rod was a woman.

The community of Ccarhuaccocco has a self-sustainable economy with livestock and agriculture by annual cycle of rainy season, the promotion of tourism to its archaeological sites motivated the visit of researchers; however, the most important variable was the situation of change in local government by women. Tourism promoted by the regional authorities does not take off because of its geographic location and distance that would improve the economy of the population. In a group interview during a work activity—the construction of vivarium that was directed by the woman Varayoc— the community members said that they did not have an exact date of the origin of the social symbolism of the position of Varayoc (or *envarado*). Quechua, the language of the Inca people that survive in the South American Andes, is the commonly spoken language in the area:

In Peru, 4,390,088 people have a native [aboriginal] language as their mother tongue, representing 16.3% of the country’s population. Of that number, 3,375,682 people (13.9%) are Quechua speakers, 444,389 (1.7%) speak Aymara as their mother language, and more than 210,000 (0.85%) speak a language from the Amazon (Diarioelperuano, 2021).

Knowledge of the local language enabled the researchers to gain easy access to the community, which is a jealous guardian of its customs through the Varayocs. Our research focused on the symbol of social control by the woman Varayoc, which broke the masculine stereotype that had persisted since the time of the ancient Incas, and has been described as follows: “The phallic symbol is the patriarchal representation par excellence (the Varayoc rod or the scepter, for example). But if it were a statue of a woman showing a tremendous vulva, it would be a scandal” (Bruce, 2022). The first woman Varayoc is Gloria Ccaico Zarate, who is a 45-year-old divorced single mother with two little children. She authorized the use of her name and personal experiences for this research. She overcame the gender stereotypes of the Andean community’s political administration regarding the transfer of power: “[…] since all Varayoc men must be married, the female partner is essential for this position, as this is how the man with the most prestige enjoys his life, and his woman participates in the honors emanating from the investiture” (Rosas and Pereyra, 2021, 109). Thus, the change in the occupant of the office of the Varayoc from men to women is important.

## 2. Methodology

This research is a field investigation that describes our observations and experiences of the customs of members of the Ccarhuaccocco community, their weekly tasks, and coordination meetings with agronomists who visit them to carry out minor projects of the state, such as specialized vivarium at high altitudes. We solicited the agreement of the entire community to participate in our study by securing the approval of the woman Varayoc, which was recorded in an official meeting (Researcher credential). We randomly interviewed members of the community of different ages to collect information. The community members habitually invite visitors to their homes to share their meals, and we enjoyed their hospitality as well. During these meals, they recounted all the events of their daily activities.

The ethnographic work allowed us to learn about the daily activities and special events of the first Varayoc woman of the community. The data collected in the observation, interview and coexistence with the community were used to analyze the recurring themes of those involved in the research. Due to the interest in promoting their archaeological sites, they are aware of the arrival of few visitors during a weekend. The person who carries the Vara radiates respect toward the community members, regardless of their age. The processing of the information is complemented with national and international literature on the transfer of power to women in equal conditions.

### 2.1. Travel to and arrival at Ccarhuaccocco

**Figure 1:**
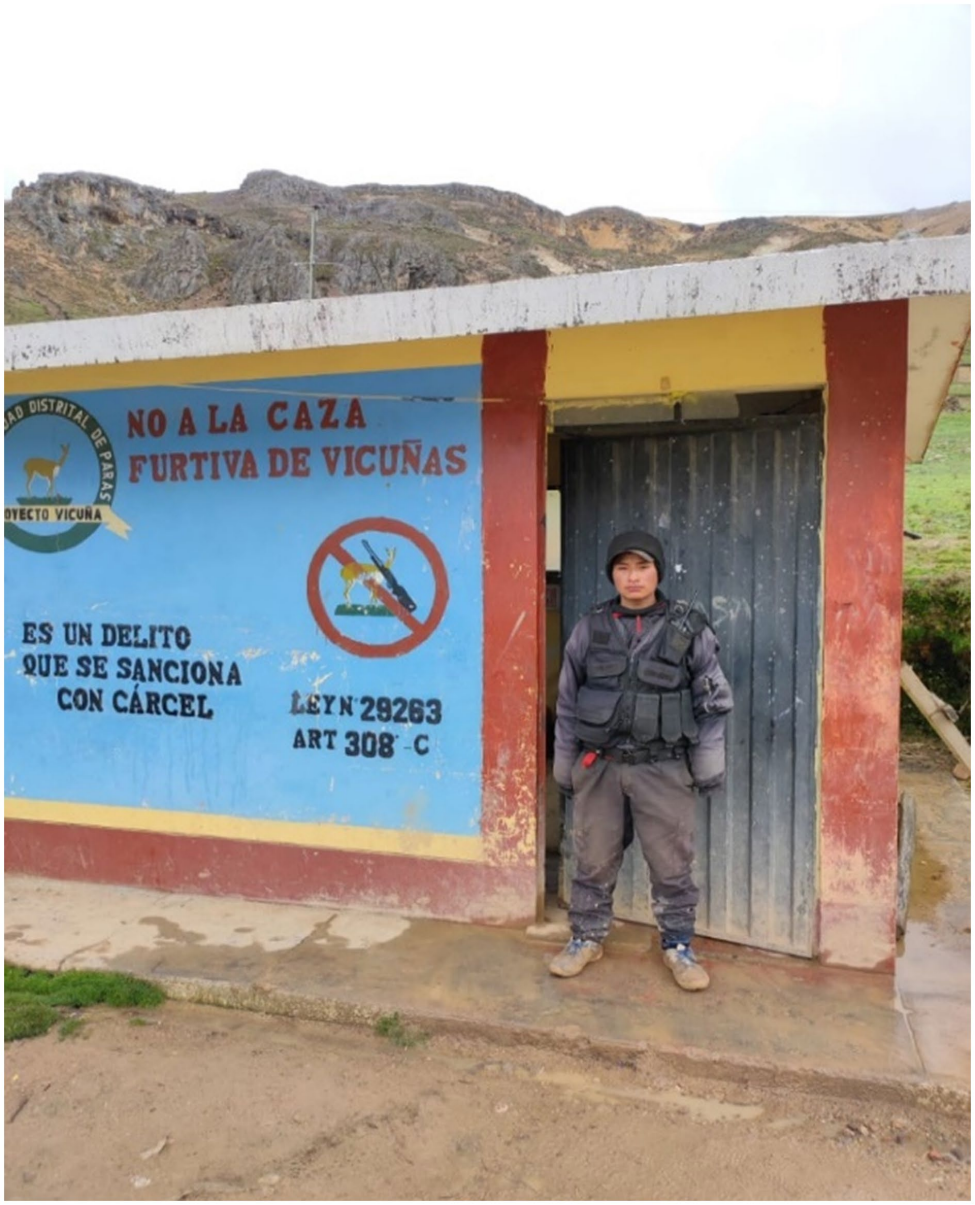
Watchman at the entrance of the district of Paras. The inscription on the wall says: “No to poaching of vicuñas. It is a crime punishable with jail. Law No. 28283, Art. 308-c”.

**Figure 2:**
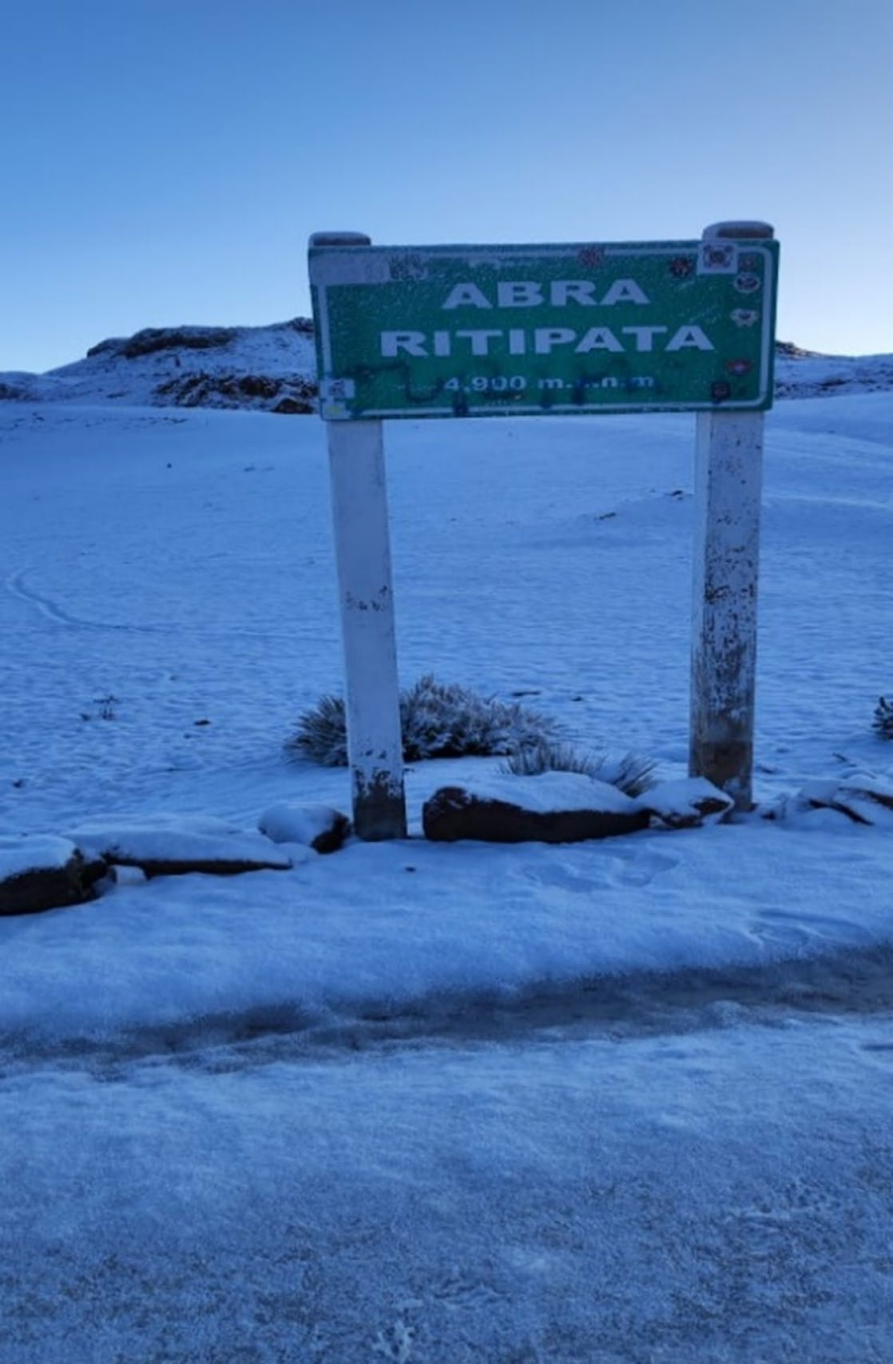
*Apu Ritipata* at the entrance of Ccarhuaccocco. Source: photographs by the authors (April 2023).

To get from Lima to Ccarhuaccocco, one has to travel on the Libertadores highway, followed by a detour to a small hamlet called Santa Fe. It has a surveillance station set-up by the District Municipality of Paras to control the passage of visitors. According to Eduardo Ccorahua, a watchman on duty, the 24-h surveillance is meant to deter rustlers, kidnappers, and thieves. The station in shown in Figure 1. When entering Ccarhuaccocco in Paras District, one immediately encounters the imposing snow-capped *Abra Ritipata,* which provides water to the area through the Ayacucho water route. This is shown in Figure 2.

The Andean plain is visible when arriving in the town of Ccarhuaccocco. One can see several kilometers of stone fences protecting crops during the summer, which is the rainy season in the Peruvian highlands. The economy of the area is basic: “Agriculture and livestock are very precarious owing to a lack of water; they [the locals] barely produce for their subsistence; therefore, there is a lot of poverty, since a majority of the adolescent and young population has migrated to the city of Lima to work” (Zambrano, 2018, 6). Dignitaries to the area visiting from other parts of Peru or from abroad are typically received by the lieutenant governor, who is accompanied by three Varayocs according to a norm going back several generations. “Without a doubt, greater access to education was a factor in the decline of the Varayoc institution” (Rosas and Pereyra, 2021, 115). Part of the lieutenant governor’s retinue was a woman Varayoc who carried the rod of command symbolizing her power in society.

Social control is evident in the different symbols carried by officials of civil authorities in the Andean communities: “The parallel fusion of Andean and Western cultural patterns is evident in the production of textiles, decoration in ceramics, details of the Varayoc’s batons or carved rods, and even the so-called *qellqas* or painted tables” (Sota, 2021).

In his interview with the researchers, the Lieutenant Governor of Ccarchuaccoco Luis Yarasca Quichca lamented the loss of power of the Varayocs due to the elimination of sub-prefects for political reasons. They were certified as political authorities of the central government: “The Ministry of the Interior today concluded the designation of 1,076 district sub-prefects at the national level, who were appointed during the administration of former president Pedro Castillo” (Ministerio del Interior, 2022). This led to a loss of their authority in the face of a growing population, and the ancestral customs of administrative symbolism in self-managed Andean communities were lost. Of course, this is not Lima’s (the central government’s) opinion of its role: “A political apparatus at the service of the regime, these positions were occupied by people committed to acting against the democratic form of governance, and with people who had relations with Movadef^2^ as well as organizations close to Sendero Luminoso”^3^ (Redaccionp21, 2022).

Political accusations against peasants of the Peruvian highlands are common, in the sense that whoever protests against the government of the day is branded a terrorists—*terruqueo*—by the Peruvian press, which is primarily owned by large corporations. In the interview, the lieutenant governor claimed that former president Pedro Castillo sought to defend local cultural identity and interests: “He treasured campaign vests from the national command of Peru Libre [his political party]—a wooden varayoc inlaid with gold metal, and a red Bolivian poncho—that received so much attention during the demonstrations” (Nacionales, 2023). Castillo was accused of motivating social demonstrations in favor of the Varayoc: “The traditional Andean rod was part of the symbolic swearing-in ceremony of the president in Ayacucho” (La República, 2021).

## 3. The symbolism of the power of the woman Varayoc

This is the first time in the history of the Ccarhuaccocco community that a woman has assumed the position of Varayoc (*envarado*): “During the entire 19th century, the institution of the Varayoc demonstrated a strong ability to summon the indigenous masses. Evidence since the beginning of the Republic speaks to the power and legitimacy of these authorities” (Rosas and Pereyra, 2021, 106). The Peruvian state recognizes the Varayocs to this day, in this area and other territories, due to their importance and international diffusion:

It is the declared cultural patrimony of the nation to the cultural manifestation called the “system of traditional authorities known as Varayoc of the District of Pisac,” Province of Calca, Department of Cusco, due to its importance, validity, and significance as a descendant of the long history of the political organization of the Andes, and as a vehicle of cohesion and identity of the population of the aforementioned district (Ministerio de Cultura, 2015).

**Figure 3:**
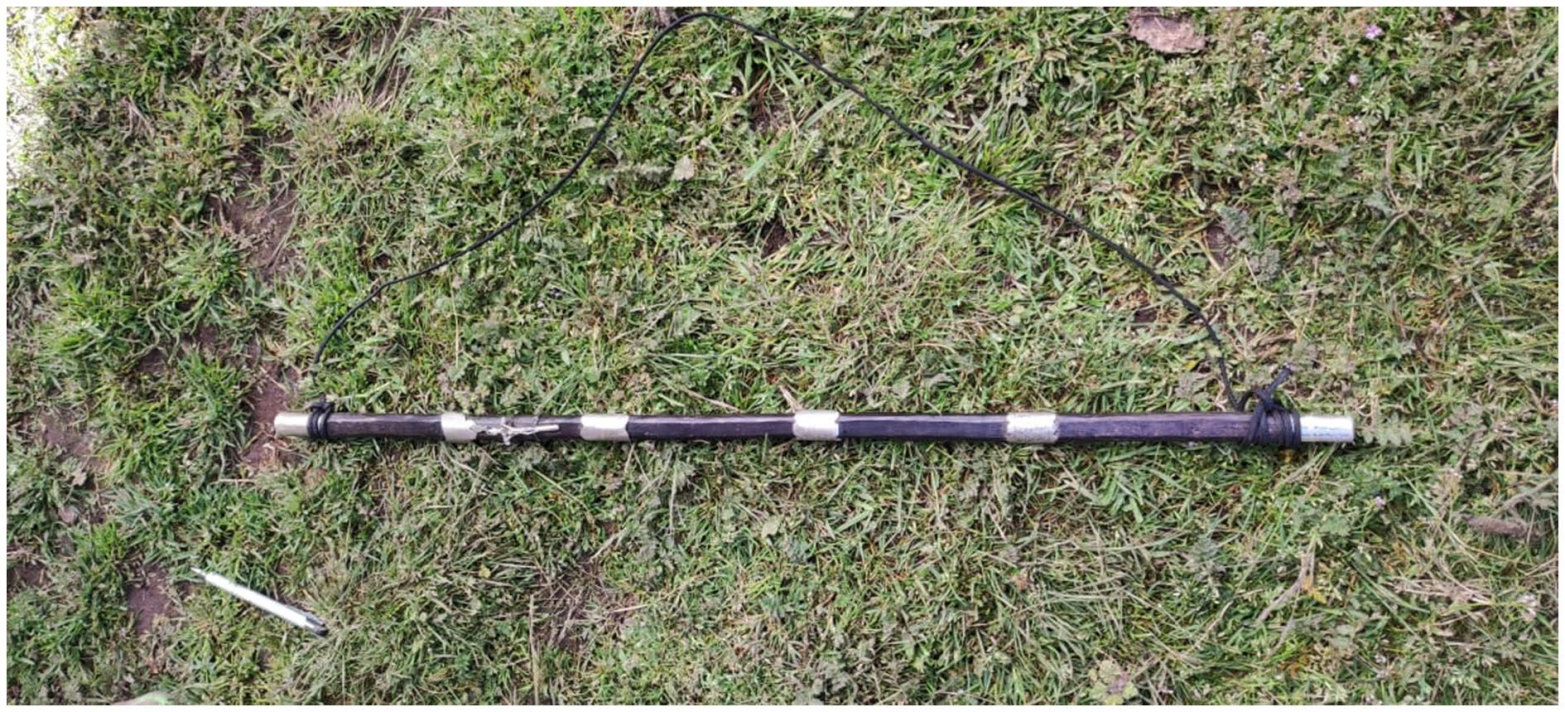
Command rod belonging to Gloria Ccaico Zárate, photographed in the backyard of her house. Source: Photography by the authors (April 2023).

Gloria Ccaico Zárate is the first female Varayoc to have held the position in the Ccarhuaccocco community. It is an active beginning of women in the life of the political administration in the opinion of Silvia Rivera Cusicanqui: “women are already actively and massively coming out into the public sphere to demand things that were previously considered exclusive to the private sphere” (Barber, 2019). The men interviewed expressed that the gradual change of having women occupy the Varayoc position is seen as a valuable opportunity to maintain the Inca Varayoc system of governance and give equal opportunities to women. She is scheduled to be in office for a year starting on January 1, 2023: “To serve as a Varayoc and climb in this network of positions involves regulation and order. The documentation lets us see a strong generational sense and the idea of trajectory in the community” (Rosas and Pereyra, 2021, 108). The woman Varayoc is in charge of 429 members who are officially registered with the community. She is fluent in Quechua, and in Spanish as a second language, in her role as an *en*var*ada* (see Figure 3).

The stick is carried as a symbol of special power for social occasions. It is made of a material called *sunchu* (common name, *Aruruhuay, sunchu;* scientific name, *Viguiera planzii Parking;* location: wild grass; use: leaves are edible)*”* (Delgado, 2004, 48). It is wood from a tree found in the Peruvian jungle. According to Gloria Ccaico, the rod she carries is a loan from an older Varayoc to exercise her symbolic authority. “‘Varayoc’ refers to the person who carries or has the rod. The word is from *vara*, meaning command rod, and *yoc*, which is the Quechua suffix that indicates possession” (Rosas and Pereyra, 2021, 105). Our interviews with locals indicated that the status of power is changing in relation to the cultural manifestations of the Peruvian highlands. There are historical records of the transfer of male power to women in different parts of Peruvian territory; it is evident that Varayoc’s rule was exclusively male. This is demonstrated by historical artistic expressions of the Peruvian territory:

The artist painted on the rods, which are traditionally used only by men who exercise some kind of authority in Andean communities—the Varayocs—and different scenes reflect the lives of women. In these scenes, she reproduces the mythology of the mermaid, as a symbol of the “virgin healthy” woman, the sexually violated woman. It also represents those who make their way in politics for the benefit of more Peruvians, and portrays the healing process of women through spirituality and their connection with the Earth (Purizaca, 2020).

**Figure 4:**
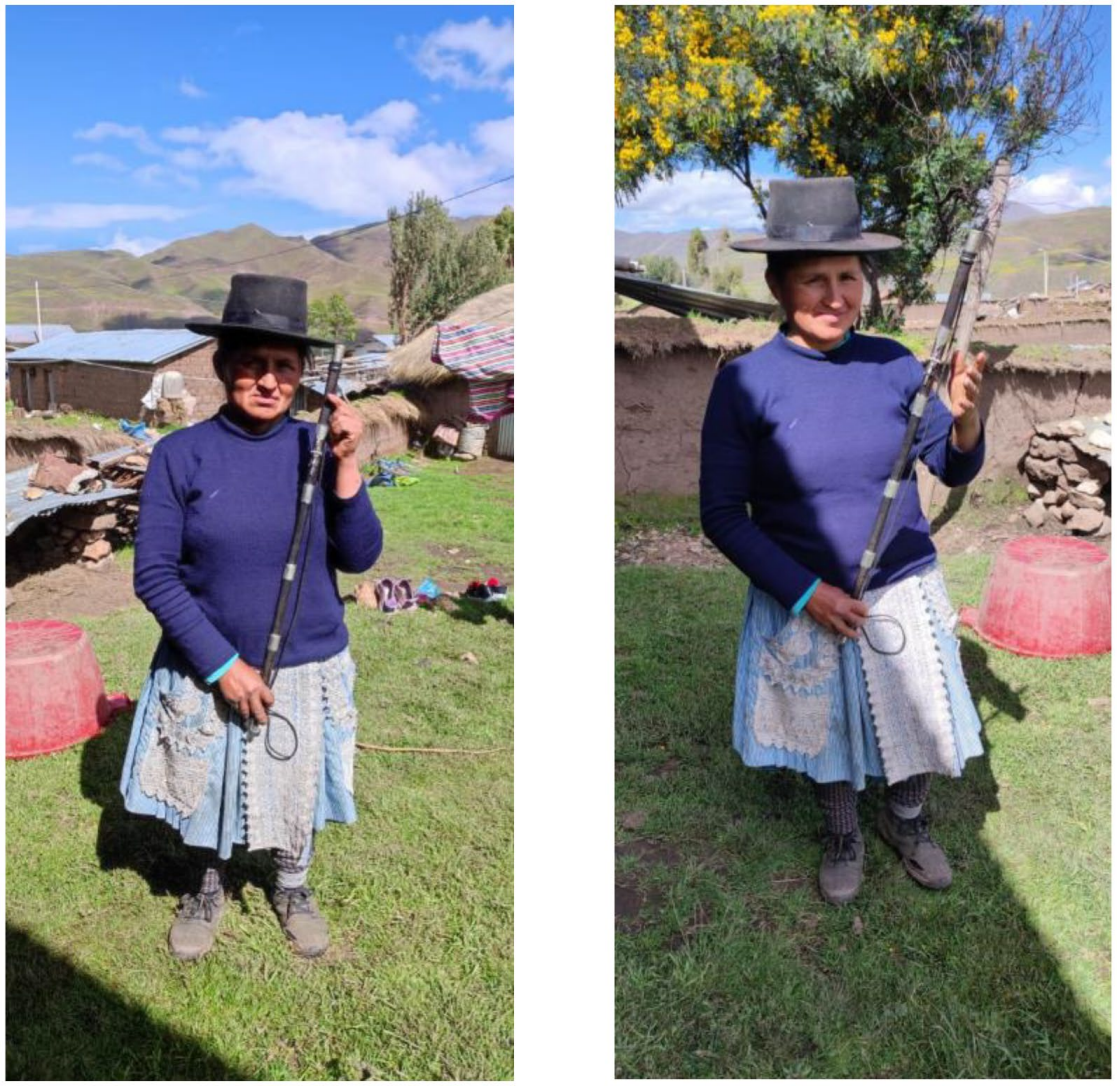
Gloria Ccaico Zárate with her command rod at her home. Source: photograph by the authors, April (2023).

The first woman Varayoc (Figure 4) commands social and political control over 429 registered community members.

In interviews, members of the Ccarhuaccocco community agreed that the appointment of a woman Varayoc was an important symbol of the change in social control, as men had held the position for hundreds of years prior:

Since the Jesuit missionary era, the town had a governor and each division had a captain, an ensign, and a militia sergeant, who brought in “infidels” to be converted. Likewise, each partiality elected a Varayoc and a mayor, who had to ensure communal work, every year, while the prosecutors had to do the same for religious offices (Ortiz, 2018, 37).

Older people in the community yearn for the past and the unquestioning obedience of the population. Now, they are resigned to changes in favor of women’s empowerment in terms of their assumption of the office of the Varayoc. They achieved this owing to their outstanding national and international work, as exemplified by the social activist Consuelo Torres Tello, who “for the third time is a candidate for the Prince of Asturias Award in the Concord category. Previously, she was decorated by the Peruvian government with the Medal of Honor and the Varayoc—a rod that represents power in the Inca Empire” (BBC Mundo.com, 2006). The rod is provided to the Varayoc so that they can carry out critical social work, and its ownership is considered an honor in Andean towns in which the system still survives. The assumption of command involves rituals typical of the community: “A commission headed by the Varayoc mayor visits the candidate family to carry out the Vara Hapichiy ceremony, of grasping the *vara,* or rod. The outgoing Varayoc asks for permission to speak and explains the reason for the visit” (Silvestres, 2006, 22). This form of command has changed radically. The assumption of command now involves social work carried out by the community member who is designated the Varayoc as a free service.

According to the records to date in Peru, Gloria Ccaico is the first woman to occupy the position of Varayoc, in other places women occupied in a symbolic way. Gloria says that she dared to propose herself in the communal board of change of command and was acclaimed by all without any objection: “These reforms involved women taking on the responsibilities of family, community and work environments to fill the gaps left by the state” (Guzmán and Triana, 2019, 28). She also affirms that there are already other candidates to succeed her when her mandate ends. The challenge to assume is a responsibility in her condition of single mother (divorced). Her condition of Varayoc obliges her to organize activities concerning the community such as the different ancestral cultural traditions maintained by the population. Gloria shares the command with two other men in the mandate; since in each year she assumes three varayocs. The two men who accompany her in the mandate manifest that the population pays more attention to the woman in her mandate. This exercise of power was demonstrated in their different daily activities of moving at all times with the Vara de mando. It was also evidenced in the most important activity of the year, the Water Festival, which lasts three days: “The Ccarhuaccocco River and is part of the Yarqa Aspiy, that is, the Ccarhuaccocco Water Festival, with a length of 45 km that runs toward the farmlands and the town of Ccarhuaccocco” (Gutiérrez-Gómez et al., 2023, 15).

### 3.1. Survival of office of Varayoc as representative of women’s power

*Envarado* (masculine) or *envarada* (feminine) in popular Peruvian culture refers to someone who enjoys the privileges that come with decision-making power. According to the Royal Spanish Academy, it is “m. and f. Peru. A person who has value or influence with an authority. m. Peru. Authority of indigenous communities whose mission is to exercise municipal functions and settle differences amicably” (RAE, 2023). The loss of credibility of male leaders in Peruvian politics has facilitated the transfer of power between genders in peasant communities, where this had previously been the exclusive domain of men: “The truth is that there is no record of the term ‘Varayoc’ in previous documentation from the 19th century. In addition, both the eras of the Tahuantinsuyo and the viceroyalty involved other positions and institutions with different functions and characteristics” (Rosas and Pereyra, 2021, 104). The Varayoc or *envarada* Gloria Ccaico from Ccarhuaccocco walks imposingly with her rod wherever she goes, and according to locals carries a *chamberin* (a whip made of cowhide) in the hidden pocket of her apron.

In her interview with the researchers, Ccaico confirmed her symbolic use of the *chamberín*, which was blessed by a priest in the village church: “Flogging with the whip is intended for the ascetic objective of ‘helping the Lord,’ and only on rare occasions has a punitive nature” (Ceruti, 2016, 19). Her neighbors, children and adults alike, have an innate respect for the *envarada* owing to the oral tradition that has been transmitted in the community from generation to generation. Ccaico said that when there is disorder during a task, meeting, community party, or in daily life, she takes out the *chamberín* and the people calm down for fear of being publicly punished. Holding office is essential to maintain social order in the community: “The community does not have fluid contact with the capital of the region and even less with the capital of Peru; its system of government is self-managed by the authorities called the Varayocs” (Gutiérrez-Gómez et al., 2023, 14).

**Figure 5:**
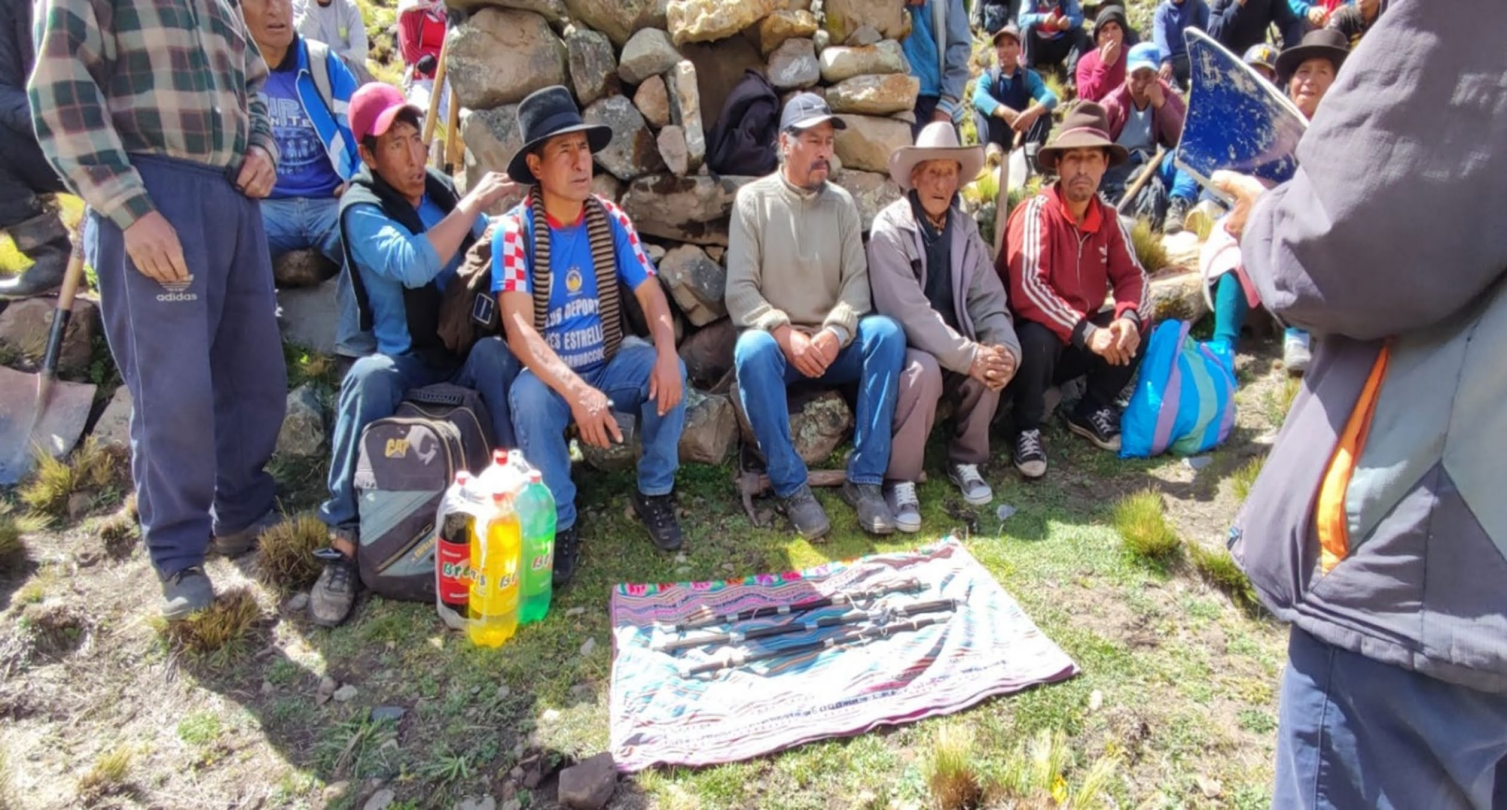
Changing of stewards for *Yarqa Aspiy* for 2024. Source: photograph by the authors (May 2023).

The tradition of carrying the rod as a symbol of power and social control is obligatory in all communal activities, for example, the rods in ceremonies. Figure 5, *Yarqa Aspiy* (cleaning of acequias) annual meeting every May 4 for three days of 24 kilometers of ditch, according to the authorities, do not have an exact start date, but estimate about 150 years of tradition infallible.

**Figure 6:**
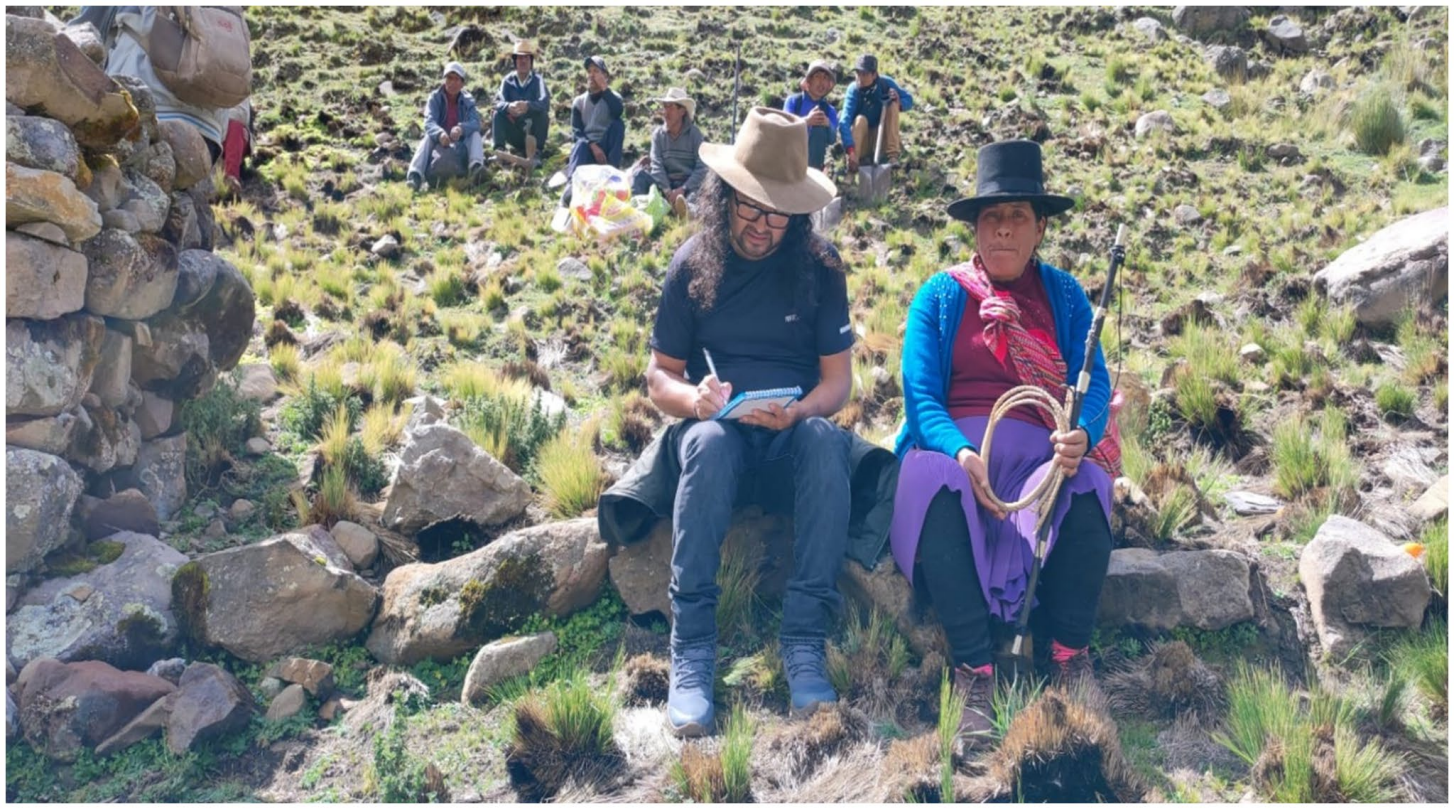
Ricardina Laurente as Varayoc on the first day of *Yarqa Aspiy*, with her *chamberin*. One of the authors takes notes beside her. Source: photograph by the authors (May 2023).

We also came across *Yarqa Aspiy* during our field work, but this is a suitable topic for another research paper. The presence of the Varayocs is essential for ceremonies related to each activity, and another person can be entrusted with the responsibility of carrying the rod if the actual holder is absent. For instance, Gloria Ccaico was unable to attend the ceremony for *Yarqa Aspiy,* and the responsibility fell to Ricardina Laurente to act as Varayoc, as shown in Figure 6.

Interviews with the community members during different activities revealed that maintaining the tradition of command requires dedication and commitment from the entire community: “San Pablo de Occo consists of three neighborhoods: Occo Centro, Ñahuinpuquio, and Matipajana. Each has its respective authorities called *campo de* var*a* [rod field] or Varayoc, who maintain order in all situations” (Zambrano, 2018, 06). Regular meetings for different community-related activities contribute to the symbolism of social control by the Varayoc: “… the [educational] institution is behind the main square; it is protected by three guards and the authorities (Varayocs), and is surrounded by family houses” (Zambrano, 2018, 07). Customary education involves teaching locals about the connection between Catholicism and the *apus* (high mountains) that sustains the survival of the Varayoc. The upper part of the Varayoc’s rod bears a silver crucifix that is related to an event in Peruvian politics and represents the religious significance of the Supreme Pontiff: “After an audience with him, President Kuczynski presented the Pope with a Varayoc (Inca command baton) as well as a replica of *Mary: Untier of Knots* (a painting from 1706 housed in Augsburg, Germany)” (Redaccionp21, 2017). This public manifestation recounts the faith of the population of the Peruvian Andes, where political modernity has not yet fully taken hold but obedience to the Varayoc is unquestioned.

The interest and curiosity of visitors as well as the patronage of such authorities as the mayor, the lieutenant governor, and the elderly in the community ensure the survival of the office of the Varayoc: “Paris held an innovative exhibition of sculptures of the Varayoc, representative of the most remote peoples of the Andes, who have created democratic systems in their villages, although the states and official governments refuse to recognize history” (Zavaleta, 2019). In addition, the Peruvian State recognizes the importance of communal governance due to its importance and transcendence: “The system of traditional authorities known as Varayoc, from the District of Písac, in the Cusco Province of Calca, was declared the cultural patrimony of the nation six years ago, with a view to making it an organic part of the state in the 2021 national bicentennial” (Zavaleta, 2019). However, this commitment by the government of the day has not materialized; on the contrary, the office of the Varayoc has since been accused of fostering terrorism.

### 3.2. Symbolism of the Varayoc in the change in political command

As in Ccarhuaccocco, the change of command in the position of the Varayoc in all Andean communities where the office still survives takes place on December 25 of each year, and the new official assumes power on January 1. The Varayoc’s rod has also been used as a symbol of power at the international level among members of the Pacific Alliance, which has express the aim “to integrate other nations, such as New Zealand, Australia, and Singapore, into the bloc. Larraín received command as Varayoc from the outgoing president, Alfonso Bustamante, who wished his successor luck in promoting the commercial block” (Economía 2019).

Members of the community confirmed a series of rituals in the ceremony signaling the change in command, such as dances at the birth of the child Jesus, speeches by the candidates for the office of Varayoc, a meeting of all the members of the community, and the blessing of the var*as* [rods] for the new *envarado*:

Its importance in the political and administrative framework in their communities has been such that its current validity is proof of this. In the same way, its distinguished emblem par excellence, the command rod, is a very present element that has had great symbolic power within the collective imagination of Peruvian society for more than half a century. This represents authority and power (Rosas and Pereyra, 2021, 103).

In the case of the study of the Varayoc woman, it generates motivation and example for other women who wish to assume the position of envarada. The tradition of assuming the Varayoc is for married people, in the case of Gloria Ccaico is divorced, the community accepts without objections. It is known in Andean history of women who only accompany their husbands: “A married Varayoc has significant help from their spouse in carrying out the protocol or the ceremonial activities of the position. As we know, the Varayoc has to organize a banquet for the entire community to celebrate taking office” (Rosas and Pereyra, 2021, 109). Despite her family situation, Ccaico took on the commitment to be the *envarada* of the town, and to comply with the requisite rituals and festivities for the change of command, such as the traditional *ccaracuy* (community snack) at Easter for the entire community.

**Figure 7:**
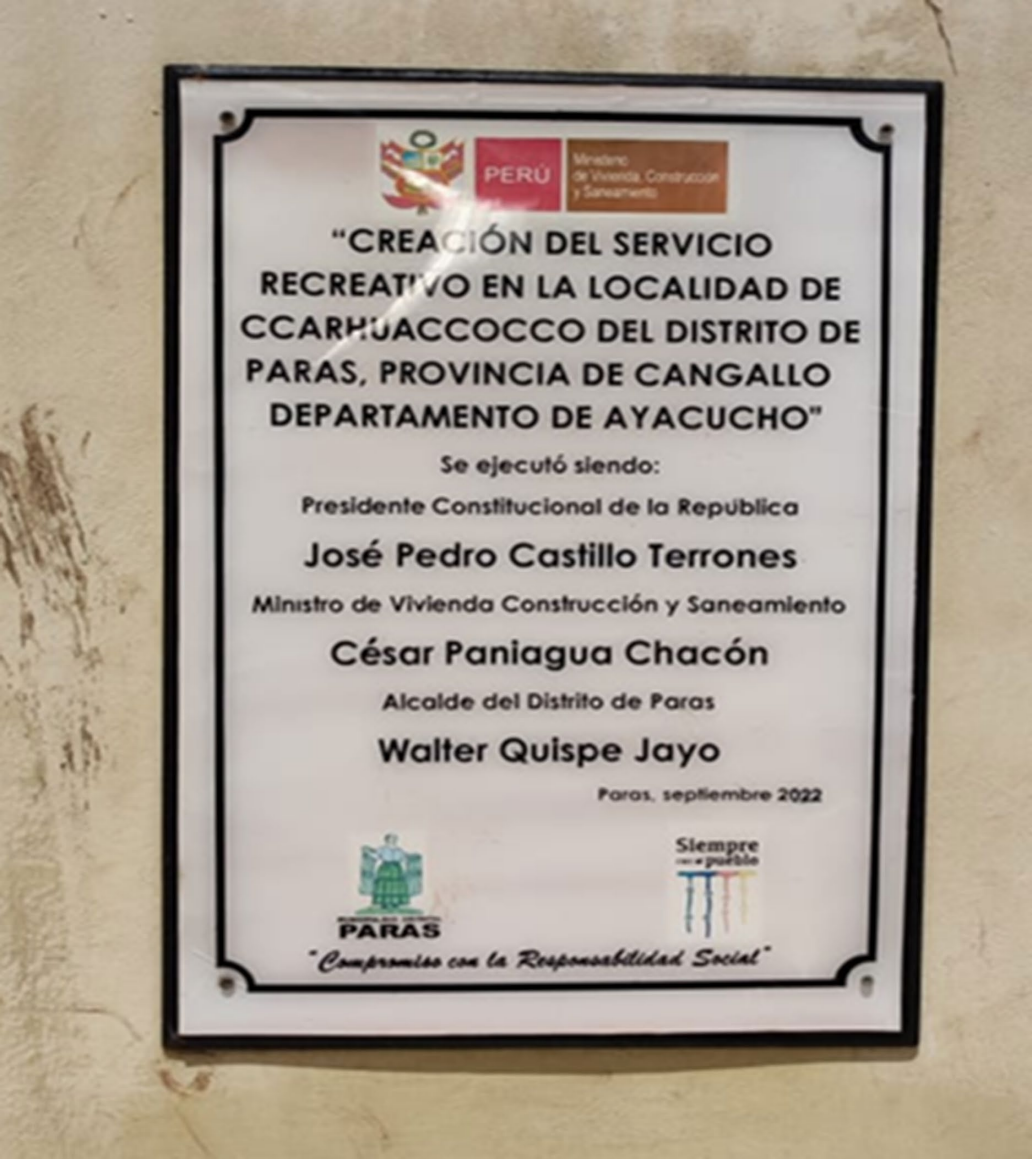
Plaque commemorating the inauguration of a recreational facility in the central plaza of Ccarhuaccoco during the administration of Pedro Castillo. Source: photograph by the authors (May 2023).

The symbolism of the power of the Varayoc and the rod of command is important for peasants of the Peruvian Andes, who identify with it to this day: “The mayor of Huamanga, Yuri Gutiérrez, gave President Pedro Castillo a typical poncho of Ayacucho, of Incan origin. Moreover, a girl gave him a Varayoc baton as a gift from the people of Ayacucho” (País informativo, 2022). The highest authorities in Peru, such as former president Castillo, became involved in the affairs of the peasants by identifying traditional power structures: “He [Castillo] received a Varayoc, a symbol of the balanced power of the Wari and the Chancas, in the historic sanctuary of the Pampa de Ayacucho, where he was sworn-in in a symbolic ceremony attended by several presidents” (Política, 2021b). The inhabitants of the Peruvian Andes identified with Castillo, whose government built a recreational facility in the central square of Ccarhuaccoco, as shown in Figure 7.

The only place of recreation for children in the community is located in the central square. The short and narrow streets of the town are paved with cement, and the streets bear the names of the 24 political regions of Peru.

**Figure 8:**
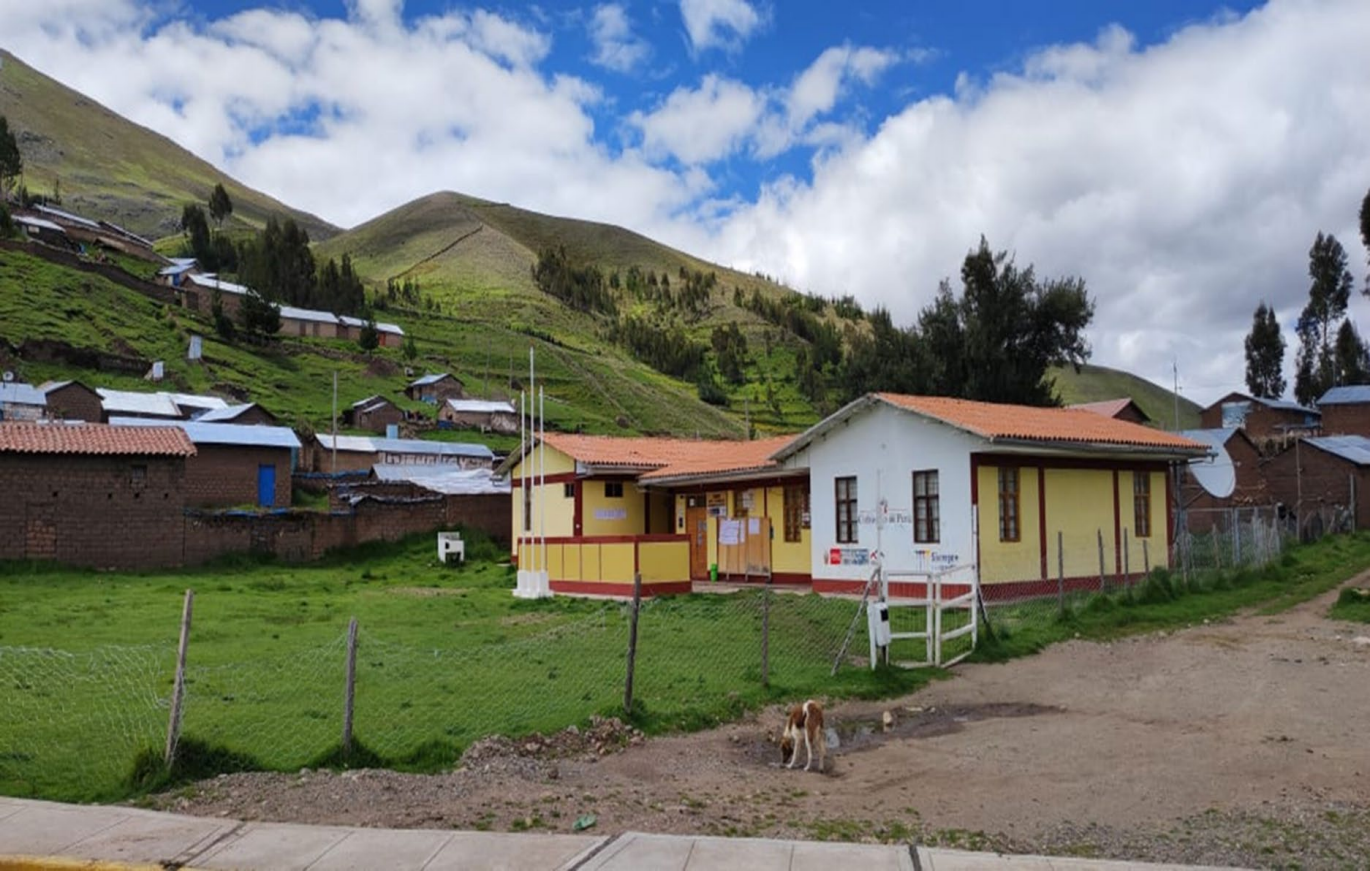
Tambo in Ccarhuaccocco, built during the government of Ollanta Humala. Source: photograph by the authors (April 2023).

Another set of facilities in the town, tambos or inns in the Andes for the benefit of visitors, were constructed during the term of former president Ollanta Humala. The administration built “76 tambos in the country, and another 101 tambos are under construction in 15 regions. By the end of 2013, the government announced that it expected to have built more than 180 tambos throughout the country” (Noticia 2013). These two facilities, the recreational center and the tambos, were the only ones that have been constructed in Ccarhuaccocco in the last few years. Former president Humala was awarded a Varayoc’s rod for this service: “The president was declared Occollo’s favorite son, and received the Varayoc from its mayor” (Huamanga 2013). The Varayoc is present during every political change in the Peruvian state and visits by state officials to Andean communities. The tambo in town currently serves as a state administrative office to host visitors, and is shown in Figure 8.

Visitors to Ccarhuaccoco are approached by the communal authorities, and the Varayoc is watchful of all strangers. Tourists have been drawn to the town due to the promotion of its cultural history by the regional government of Ayacucho. They visit the local archaeological center and the snowy *Riti Pata*, witness local farmers at work, and enjoy the hospitality of the Varayoc and local inhabitants.

### 3.3. The Varayoc as a symbol of recognition of political power

The political power of the state is limited in the Peruvian Andes, because of which the office of the Varayoc remains active today: “The director of the BNP [National Library of Peru] gave the historian a Varayoc and a scroll. Pablo Macera [Renowned historian] thanked the BNP for its hospitality” (Redaccionp21, 2015). This relation influences the importance of the command rod in the Andean communities of Peru: “Pedro Castillo received the Varayoc and a typical poncho. The objects were delivered by the mayor of Huamanga to welcome him” (Política, 2021b). The Varayoc represents power: “President Pedro Castillo received the Varayoc—a symbol of power—in the historic sanctuary of the Pampa de Ayacucho, where he will take a symbolic oath” (Política, 2021a).

The peasant communities of the Peruvian Andes are self-managed, and the Varayoc plays a leading role in social control. In the absence of equivalents in the Spanish language, the symbols that represent this power facilitate respect for the authority of the command rod of the *envarado* or, in the present case, the *envarada*: “On the other hand, the biologization of gender involves associating the feminine ‘power’ with the uterus and the masculine with the phallus” (Cordovil, 2015, 445). This a generational advance. On the contrary, the media exalts the historical sexism in Peruvian culture: “The public user considers feminism and machismo to be part of the spectacle in Peru, which is part of the gossip of everyday life” (Gutiérrez and Munaris, 2023, 02). Women in the Andean community of Peru are finally assuming their rightful place as part of society.

The most important gift that national politicians receive when they visit the Andean communities of Peru is the traditional rod/baton of command as a symbol of social control:

… [Antauro Humala] the ethno-Cacerist^4^ leader visited the town center of San Martín de Porres in the Ancahuasi district, the place where President Pedro Castillo was present a week ago. In this area, a local representative gave the “key” to the city, or “Varayoc,” to Humala. This same ceremony was performed with … the president a few days ago (Quispe 2022).

The rulers of Peru know the importance of the command of the rod, and receiving it from local authorities as administrators of the social order is fundamentally important. Moreover, “different regions of the country [feature] adaptations of other authorities linked to the structure of the government of the Varayoc, with particularities” (Silvestres, 2006, 25). This symbolism of recognition of the Varayoc by the political center of Lima generates pride and symbolic value for the peasants, who respect the traditional *envarado*. Women have limited participation in presenting symbolic gifts to visiting dignitaries. These manifestations of public recognition of the political authorities is important in Ccarhuaccoco because they participate in matters related to local development.

As mentioned above, women rarely participated in the ceremony marking the change of command as *envarada*, and their typical role was that of wife and partner. Assuming Varayoc’s critical position made the man (since the occupant was, until recently, always a man) commit his entire family to the community, an example of female participation in the position as secondary:

… the wife stated that they still do not have enough goods to pass the charge of the Varayoc. She needs to spin, weave llikllas and ponchos, and to sow fields and slaughter animals, at the insistence of the visitors, on the condition that everything is done in *ayni*^5^ (Silvestres 2006, 22).

This study verified the transformation brought about by the acceptance of women in Andean communities as bearers of political power as Varayocs. Our interview with the first woman Varayoc from Ccarhuaccocco showed that there is potential for more women to assume the office, and names of women have been mooted for the position for 2024.

## 4. Conclusion

A generations-old male-dominated political system persists in the Peruvian Andes. The incipient acceptance of women to the office of the Varayoc is a radical change in this context as it overcomes the barriers of sexism of the past. The lack of research on women Varayoc in the Peruvian Andes limits our knowledge of this transfer of power: “The limited number of investigations creates the need to systematize studies on Varayocs and delve into the nature of this institution” (Rosas and Pereyra, 2021, 104). The Peruvian state recognizes the organizational role of the Varayoc in Andean communities. However, this lacks an official structure, especially with regard to the recognition of women as part of Andean society with equal opportunities as those afforded to men.

It is important to overcome the prejudicial idea that the rod of command is representative of masculine physiology, and participation in offices of authority is thus forbidden for women. In our field visit to the Andean community of Ccarhuaccocco, we witnessed local people involved in different activities of daily life, including chores, meetings, receiving visitors, and organizing festivals to honor patron saints. We found that the locals held the woman Varayoc in high esteem and proudly acclaimed her.

The lack of knowledge of the exact period of origin of the Varayoc as a local authority in Andean communities does not detract from the importance of the office, which has recently begun accepting women, as a vehicle of social control. Oral culture in the Quechua language is essential in the absence of written records in the Andean community: “A mention will be made of oral history in interviews with residents of the Acopia District in Acomayo Province, Cusco. They keep their memory alive, the memory of ancient and venerable Varayocs” (Rosas and Pereyra, 2021, 105). The recognition of Quechua as the official language of communication in the region facilitates the oral transmission of customs from one generation to another, including lessons of the importance of carrying the rod of command.

The power of the rod, a symbolic Andean instrument of command, has been exhibited nationally and internationally—for instance, in the Peruvian capital of Lima when a transfer of power takes place: “López Aliaga also received the Varayoc, which is a traditional Andean rod used in the Inca heyday” (Mariscal, 2023). Symbol of the Lima mayoral handover ceremony, this candidate made racist statements to the peasants, as is the case of the presidential candidate who considers himself Andean: “Former presidential candidate Rafael López Aliaga, together with other politicians, called for the death of candidate Pedro Castillo, from [the political left-wing party] Peru Libre, at a public meeting he organized, in favor of the candidacy of Keiko Fujimori from Fuerza Popular [a right-wing party]” (Política, 2021c). The Peruvian state and political organizations need to recognize the importance of the advent of the woman Varayoc in Andean territories of the country.

## 5. Discussion

The scenario of Andean communities marked by the predominance of male power is changing substantially with the transfer of power to women. The symbolism of the Inca government, still present today, is being transferred to women in markedly macho scenarios. Generational change is fundamental among young people; the community members consider the transfer of leadership to women to be essential: “Attempts to communicate the essence of the problem in interpersonal relationships in a society marked by machismo and the supposed vigilance of the prototype morality of social guidance” (Gutiérrez-Gómez, 2022, 170). In some communities there is a complex of belittling women, who are unable to take on the challenge of controlling and governing the peasants, expressing that they will not listen to their mandates. The research work is important to demonstrate the change of ancestral power designed for men, directly related to the phallus. The belief must change, that is the contribution that is given to the world society where machismo prevails in power.

In the search for information, no example of this change of command of the Varayoc symbolism was found, which is the original contribution of the research work. During the data collection process, the authorities informed in a special meeting about our research, this could have been our research limitation. The villagers could have agreed to act with dissimulation; however, we analyzed our observation in different scenarios of the daily life of the first Varayoc woman. For future research, our observation and coexistence with the villagers yields important data, that more women want to assume the position; because they are seen as powerful and are interested in the international community about their feminine role in power in the Andes.

## Data availability statement

The original contributions presented in the study are included in the article/supplementary material, further inquiries can be directed to the corresponding author/s.

## Ethics statement

Ethical approval was not required for the studies involving humans because the authorities of the town were consulted for the work, and the research was carried out with authorization and a researcher’s credential from the Universidad Nacional Autónoma de Huanta. The studies were conducted in accordance with the local legislation and institutional requirements. The participants provided their written informed consent to participate in this study. Written informed consent was obtained from the individual(s) for the publication of any potentially identifiable images or data included in this article.

## Author contributions

EG-G contributed to the conception and design of the study in the community. JH-A and EG-G contributed to annotation management, data cleaning, and maintenance of research data. AQ-A contributed to the initial draft and revision of the final document. RG-R contributed to the critical review, the commentary and the phases prior to the final drafting. YH-S contributed to the interview in Quechua, active participation in community tasks and meetings. All authors contributed to the article and approved the submitted version.

## Footnotes

^1^A man with influence who bears, in this particular case, a rod.

^2^*Movimiento por la Amnistía y Derechos Fundamentales* [Amnesty and Fundamental Rights Movement] is an organization linked to the Shining Path.

^3^The Shining Path is a Peruvian Maoist communist political party that used guerrilla tactics and violent terrorism in the 1980s.

^4^A Peruvian ethnic nationalist movement that exalts Andrés A. Cáceres, a military hero against the Chilean invasion of Peru in the 19th century.

^5^Reciprocal or mutual work among members of Andean communities.
